# Simultaneous multitarget radiotherapy using helical tomotherapy and its combination with sorafenib for pulmonary metastases from hepatocellular carcinoma

**DOI:** 10.18632/oncotarget.9374

**Published:** 2016-05-14

**Authors:** Taiwei Sun, Jian He, Shumin Zhang, Jing Sun, Mengsu Zeng, Zhaochong Zeng

**Affiliations:** ^1^ Department of Radiation Oncology, Zhong Shan Hospital, Fudan University, Shanghai, P.R. China; ^2^ Department of Radiology, Zhong Shan Hospital, Fudan University, Shanghai, P.R. China

**Keywords:** hepatocellular carcinoma, pulmonary metastases, prognosis, radiotherapy, helical tomotherapy

## Abstract

We evaluated radiotherapy using helical tomotherapy (HT) combined with sorafenib for treatment of pulmonary metastases from hepatocellular carcinoma (HCC). We also analyzed potential prognostic factors and further validated the combination treatment. The objective response rate in the total cohort of 45 patients treated with HT (with or without sorafenib) was 66.7% (complete response, *n* = 1; partial response, *n* = 29), with no adverse events > grade 2 in severity. Median progression-free survival (PFS) and overall survival (OS) were 7.50 ± 0.53 and 26.40 ± 2.66 months, respectively. The addition of sorafenib was associated with increased PFS (11.80 ± 1.55 *vs* 5.80 ± 0.52 months, *p* = 0.006) and increased OS (29.60 ± 5.23 *vs* 21.90 ± 5.17 months, *p* = 0.007). After multivariate adjustment, the risk of disease progression associated with combination treatment was significantly lower (*p* = 0.022) compared with HT only, and survival was significantly longer (*p* = 0.014). Further validation confirmed the benefit of combination treatment. Prognostic factors were number of pulmonary metastases for PFS (19.00 ± 7.15 months for ≤3 lesions *vs* 5.80 ± 0.26 months for >3 lesions, *p* < 0.001) and intrahepatic tumor status for OS (28.50 ± 2.76 months for well-controlled tumors *vs* 15.60 ± 6.38 months for uncontrolled tumors, *p* = 0.011). In conclusion, radiotherapy with HT for pulmonary metastases is feasible without major complications, and its combination with sorafenib may be a promising approach in a subgroup of patients.

## INTRODUCTION

Hepatocellular carcinoma (HCC) is one of the most commonly occurring malignant tumors worldwide, with extrahepatic metastases primarily affecting the lungs [1]. Radiotherapy is a preferred treatment choice for local control of metastatic lesions. We previously reported good palliative outcome and safety with three-dimensional conformal radiation therapy (3D-CRT; ≤ 60 Gy) in a series of 13 patients with pulmonary metastases from HCC [2]; however, multiple pulmonary lesions are difficult to treat with 3D-CRT. Helical tomotherapy (HT), a type of intensity-modulated radiotherapy, is a novel technique that can provide conformal dose delivery by using image-guided radiotherapy.

Sorafenib is an orally bioavailable multitargeted tyrosine kinase inhibitor with potential antiangiogenic and antiproliferative properties, which acts by blocking a number of protein kinases. This drug prolongs survival and the time to progression in patients with advanced HCC. Sorafenib is also effective in patients with extrahepatic spread, especially pulmonary metastases [3].

Currently, the information regarding treatment of pulmonary metastases from HCC is limited. We, therefore, evaluated treatment outcomes of HCC patients with pulmonary metastases who received HT in combination with sorafenib, as well as important prognostic factors for progression-free survival (PFS) and overall survival (OS).

## RESULTS

### Patient characteristics

A total of 45 patients with pulmonary metastases from HCC were included in this study. The study population was predominantly male (84.4%). Most patients had viral hepatitis (93.3%), and most metastatic lesions were ≤ 3 cm (75.6%). At the start of HT therapy, liver function was classified as Child-Pugh A for 44 patients (98%). In one patient (2%) with Child-Pugh B status before treatment, liver function improved and was classified as Child-Pugh A 1 week after the start of HT therapy.

### Response to radiotherapy

**Figure 1:**
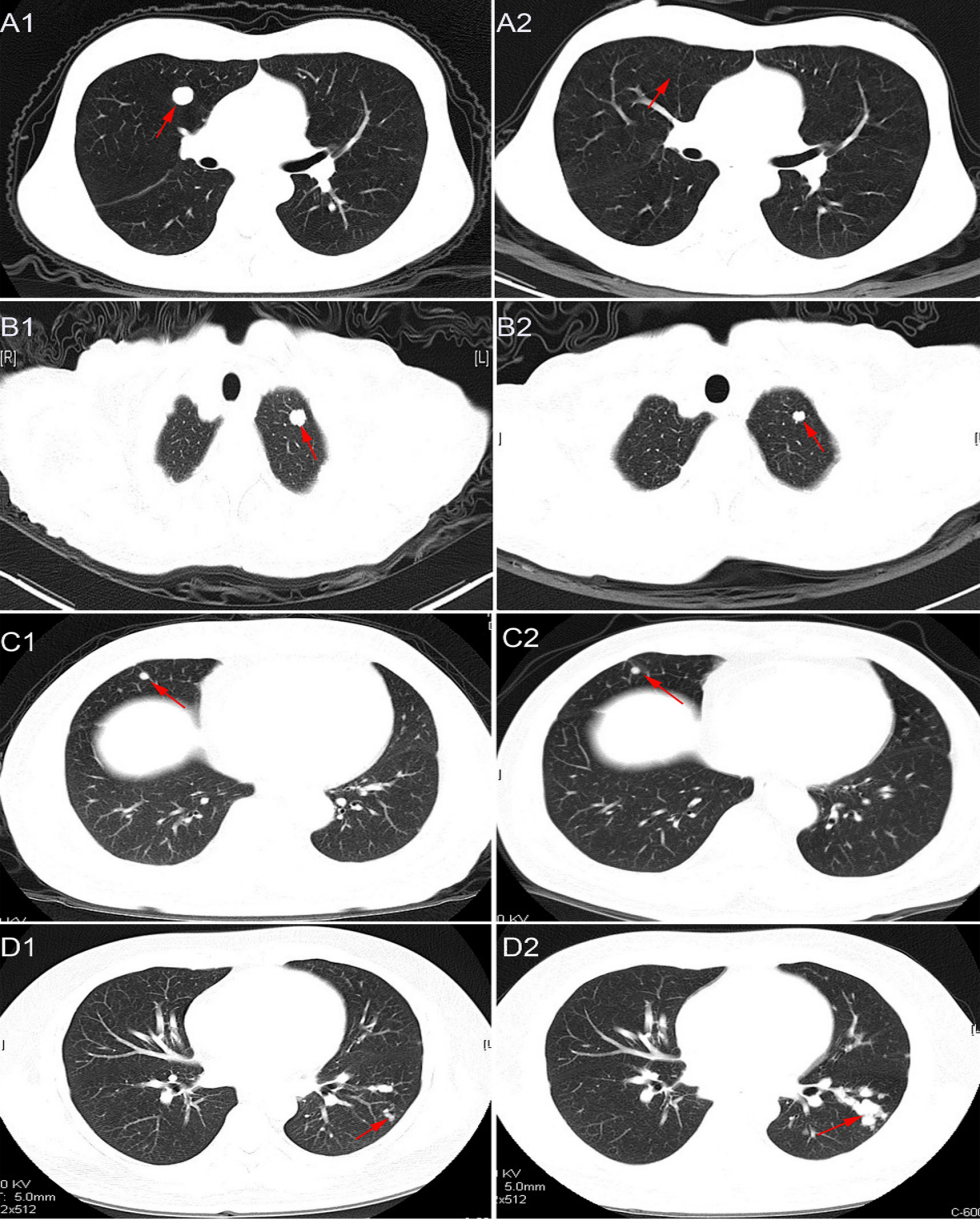
Representative images of chest CT scans showing response to radiotherapy CR: **A1.**, pre-radiotherapy; **A2.**, post-radiotherapy. PR: **B1.**, pre-radiotherapy; **B2.**, post-radiotherapy. SD: **C1.**, pre-radiotherapy; **C2.**, post-radiotherapy. PD: **D1.**, pre-radiotherapy; **D2.**, post-radiotherapy.

Tumor response to radiotherapy is presented in Figure 1. Complete response (CR) was achieved in 1 patient (2.2%), partial response (PR) in 29 patients (64.4%), stable disease (SD) in 14 patients (31.1%), and progressive disease (PD) in 1 patient (2.2%). The objective response rate (CR + PR) in the total cohort was 66.7%. A total of 195 pulmonary metastatic lesions were detected in the 45 patients. After radiotherapy, CR was achieved in 13 lesions (6.7%), PR in 137 lesions (70.3%), SD in 38 lesions (19.5%), and PD in the remaining 7 lesions (3.6%), for an objective response rate (CR + PR) of 76.9%. No significant relationship was observed between response to radiotherapy and clinicopathologic features such as gender, age, and number of pulmonary metastases (Supplementary Table S1).

### Survival outcomes

**Figure 2:**
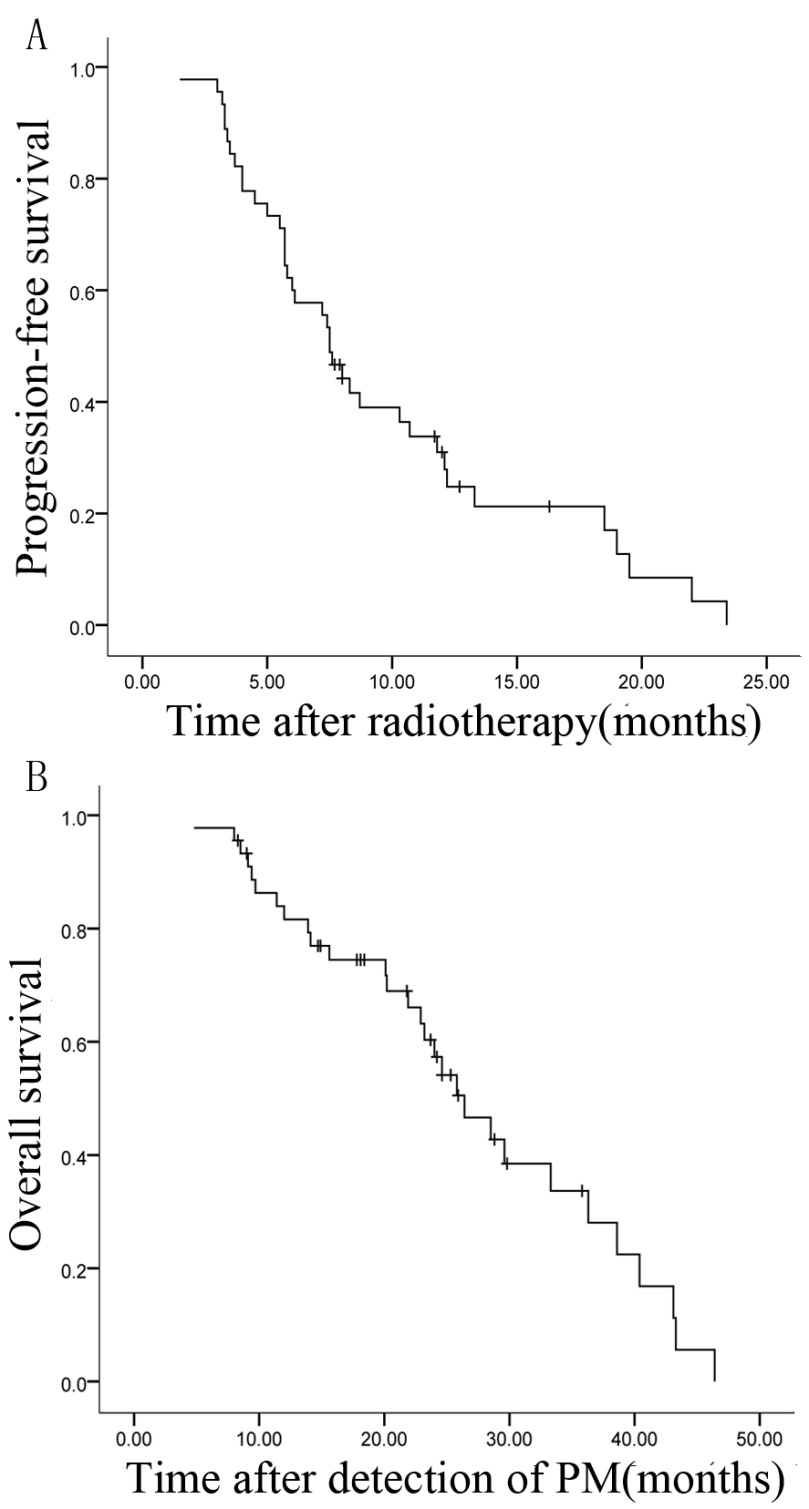
Kaplan-Meier survival curves of 45 patients with pulmonary metastases (PM) from HCC **A.**, **B.** PFS and OS after diagnosis of pulmonary metastases.

Median PFS for all patients was 7.50±0.53 months (95% CI, 6.47-8.53 months). Median OS after diagnosis of pulmonary metastases was 26.40±2.66 months (95% CI, 21.19-31.61 months). The 2-year survival rate after diagnosis of pulmonary metastasis was 46.7%. Survival curves are shown in Figure 2A and 2B.

### Factors affecting treatment outcomes

**Figure 3:**
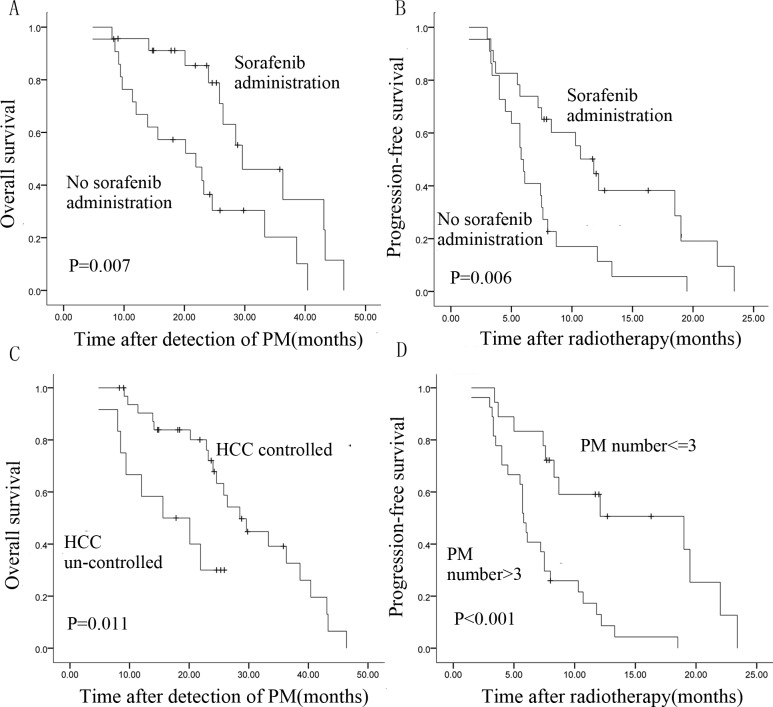
Kaplan-Meier survival curves of patients with pulmonary metastases from HCC **A.**, **B.** OS and PFS patients treated with HT only or combination treatment (HT and sorafenib). **C.** OS is associated with intrahepatic tumor status. **D.** PFS is associated with number of pulmonary metastases (log-rank test).

Univariate analysis revealed that patients who received HT only were more likely to experience cancer progression (*p* = 0.006) and death (*p* = 0.007) compared with those receiving combination treatment (HT and sorafenib) (Table 1). For patients receiving HT only, the rates of PFS and OS were 45.5% and 66.8%, respectively, at 1 year following radiotherapy, and 0% and 30.4% at 2 years. However, for patients receiving combination treatment, the rates of PFS and OS were 55.2% and 91.1%, respectively, at 1 year, and 0% and 78.8% at 2 years. Kaplan-Meier curves comparing survival of patients receiving HT only with that of patients receiving combination treatment are shown in Figure 3A and 3B. After multivariable adjustment, the risk of disease progression was found to be significantly increased in patients receiving HT only (hazard ratio [HR], 2.23; 95% CI, 1.12-4.42; *p* = 0.022), and survival was shorter in this group (HR, 2.79; 95% CI, 1.23-6.33; *p* = 0.014; Table 1).

**Table 1:** Univariate and multivariate analyses of prognostic factors associated with progression-free survival or overall survival among 45 patients

Parameter		PFS				OS				
		Univariate *P*	Multivariate			Univariate *P*		Multivariate		
			HR	95%CI	*P*		HR		95%CI	*P*
Gender										
	Male *vs* Female	0.418			NA	0.122				NA
Age										
	Age <60 *vs* Age≥60	0.704			NA	0.578				NA
PDR(months)										
	≥6 *vs* PDR < 6	0.418			NA	0.123				NA
PmFI(months)										
	≥12 *vs* <12	0.423			NA	0.297				NA
AFP of pre-RT										
	≥20 *vs* <20	0.720			NA	0.750				NA
Viral hepatitis										
	Present *vs* Absent	0.679			NA	0.562				NA
Number of metastases										
	*n*≤3 *vs n*>3	<0.001	3.76	1.66-8.49	0.001	0.406				NA
Maximum size of the metastatic lesions(cm)										
	≤3 *vs* >3	0.206			NA	0.151				NA
Intrahepatic tumor										
	Active *vs* Inactive	0.652			NA	0.011	2.92		1.30-7.30	0.024
Therapeutic models for liver tumor										
Resection *vs* TACE *vs* Liver transplantation		0.812			NA	0.693				NA
Sorafenib										
	No *vs* Yes	0.006	2.23	1.12-4.42	0.022	0.007	2.79		1.23-6.33	0.014
ECOG										
	0-1 *vs* 2	0.603			NA	0.924				NA
Other metastasis except lung										
	No *vs* Yes	0.383			NA	0.099				NA

Abbreviations: PFS, Progression-free survival time; OS, overall survival. PDR, period from the detection of pulmonary metastases to radiotherapy; PmFI, Pulmonary metastases-free interval; AFP, α-Fetoprotein; TACE, Transarterial chemoembolization; ECOG, Eastern Cooperative Oncology Group.

Results of univariate analyses indicated that the number of lung lesions and intrahepatic tumor status were significant prognostic factors for PFS and OS, respectively (median PFS according to number of lung lesions, 19.00 ± 7.15 for ≤3 lesions *vs* 5.80 ±0.26 months for >3 lesions, *p* < 0.001; median OS according to intrahepatic tumor status, 28.5 ± 2.76 *vs* 15.60 ± 6.38 months, *p* = 0.011). Survival curves are shown in Figure 3C and 3D. Results of multivariate analysis confirmed that multiple lesions and uncontrolled HCC were significantly associated with poorer outcomes (Table 1). Patients with > 3 lesions were approximately 3.5 times more likely to experience tumor progression (HR, 3.76; 95% CI, 1.66-8.49; *p* = 0.001) than patients with ≤ 3 lesions. Uncontrolled intrahepatic disease was associated with a high risk of death (HR, 2.92; 95% CI, 1.30-7.30; *p* = 0.011).

### Adverse events

Treatment tolerability was analyzed by comparing adverse events (AEs) between patients who received sorafenib and those who did not. As shown in Table 2, six patients receiving combination treatment and nine receiving HT only experienced radiation esophagitis or pneumonitis. In the combination treatment group, most of the AEs were related to sorafenib (increased levels of aspartate transaminase and alanine transaminase, *n* = 4; hand-foot skin reaction, *n* = 3; anorexia, *n* = 3; and diarrhea, *n* = 2). None of AEs were > grade 2. In patients who received HT only, no drug-related AEs were observed, except for one case of anorexia. Bone marrow suppression was observed in one patient (i.e., leukocytopenia and thrombocytopenia).

**Table 2:** Comparison of adverse effects among patients with or without sorafenib administration

	No sorafenib	Sorafenib
Total (n)	12	17
Radiation esophagitis(Grade I-II)	3	1
Radiation pneumonitis(Grade I-II)	6	5
AST/ALT increased	0	4
HFSR	0	3
Anorexia	1	3
Diarrhoea	0	2
Hypertension	0	0
Anemia	0	0
Leukocytopenia	1	0
Thrombocytopenia	1	0

Abbreviations: AST, aspartate aminotransferase; ALT, alanine aminotransferase; HFSR, hand-foot-skin reaction.

### Further validation of combination treatment

**Figure 4:**
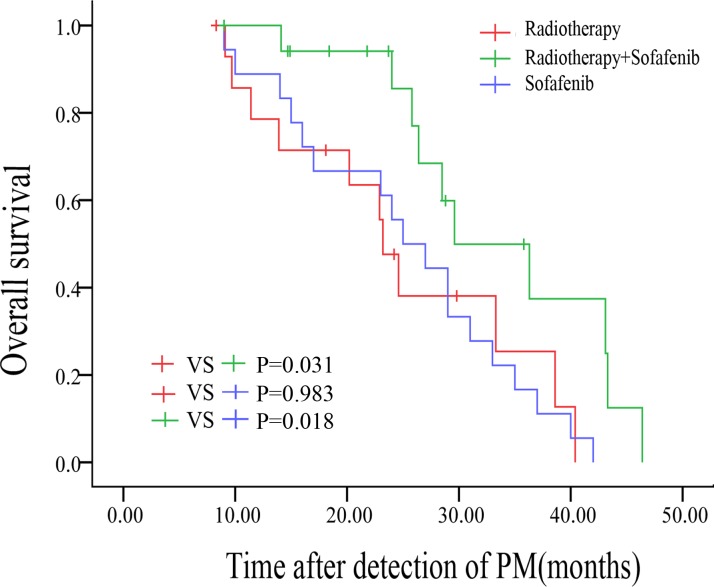
Kaplan-Meier survival curves of patients who received radiotherapy or sorafenib or both (compared by log-rank test)

The median OS was 23.20 ± 1.35 months for patients receiving radiotherapy only (Rt group), 25.00 ± 3.18 months for patients receiving sorafenib only (S group), and 29.60 ± 5.17 months for patients receiving combination therapy (HT and sorafenib, Co group) (Rt *vs* Co, *p* = 0.031; S *vs* Co, *p* = 0.018; Rt *vs* S, *p* = 0.983). Survival curves for these treatment groups are shown in Figure 4.

## DISCUSSION

Advancements in therapeutic modalities such as surgical resection, radiofrequency ablation, and transcatheter arterial chemoembolization have improved the prognosis of patients with HCC. Nevertheless, the prognosis of patients with extrahepatic metastases remains poor [4, 5]. Pulmonary metastases are diagnosed in as many as 34.5% of patients with HCC [6]. HCC is generally chemoresistant, and outcomes of systemic therapy (e.g., survival rate) have been disappointing [7]. In most cases, interventional chemotherapy and pulmonary metastasectomy are contraindicated for patients with multiple metastases. HT is a treatment option for such challenging cases, enabling simultaneous multitarget radiotherapy without increasing toxicity.

In the present study the objective tumor response in the total cohort was 66.7%, which is consistent with previous studies [8]. Although our previous studies with 3D-CRT achieved a higher response rate (76.9%), the cohort was small (13 patients), and the patients had fewer lesions [2]. In the present study, we observed a 76.9% response rate for the 195 metastases, suggesting that patients with fewer metastatic lesions may achieve a higher tumor response rate. In addition, most of the AEs observed in this study were related to sorafenib and were transient and mild to moderate in severity. None of the patients discontinued HT prematurely or required continued therapy.

In this study, patients treated with HT (with or without sorafenib) had a median PFS of 7.5 ± 0.53 months and median OS of 26.4 ± 2.66 months. Kitano et al. [9] reported similar survival rates for patients with pulmonary metastases from HCC who underwent pulmonary metastasectomy. However, in that study most patients had fewer than three lesions, whereas most of the patients in the present study had > 3 lesions.

To date, sorafenib is the only drug shown to increase OS in patients with advanced or metastatic HCC [10–11]. Li et al. reported that ^125^I brachytherapy combined with sorafenib was safe and feasible in patients with multiple lung metastases from HCC [12]. Herein, we demonstrated that sorafenib administered during and after HT in patients with lung metastatic lesions was more beneficial than HT alone. The PFS rate at 1 year following radiotherapy was higher for patients receiving combination treatment compared with those receiving HT alone (55.2% *vs* 45.5%). This difference may be attributed to the effectiveness of sorafenib on microscopic lesions outside the field of irradiation, as lung metastasis often gives rise to multiple microscopic lesions that lead to subsequent recurrence and metastases [2]. Results of univariate and multivariate analyses indicated that the addition of sorafenib to HT improves OS. This finding was confirmed in a further validation cohort, in which combination treatment was found to be more beneficial than HT or sorafenib alone.

We found that local control was significantly better in patients with ≤ 3 pulmonary lesions than in those with > 3 pulmonary lesions (*p* < 0.001), which is consistent with previous studies reporting that the number of metastatic lesions is a preoperative prognostic factor [13–15]. Results of univariate and multivariate analyses confirmed this association between lesion number and survival; however, these results were obtained from the total cohort that underwent HT (with or without sorafenib), and the association was significant only for PFS. This finding suggests that the presence of > 3 lesions indicates more aggressive metastasis that is more difficult to control with the same therapeutic dose. The presence of intrahepatic tumors was another prognostic factor, indicating that incomplete control of intrahepatic HCC lesions is associated with poorer survival. A previous study reported that surgical resection of intrahepatic HCC tumors was associated with better survival compared with HCC controlled by local treatment such as ablation, interventional radiology, or ethanol injection [15].

Although several small studies have described surgical resection of oligometastases from HCC [9, 13, 14], the indications for these procedures have not yet been standardized. More importantly, no published studies have compared treatment outcomes between resection and image-guided radiotherapy for lung metastases from HCC. In this present study we evaluated the outcomes of patients treated with HT only *versus* combination treatment (HT and sorafenib), patients with ≤ 3 *versus* > 3 lung lesions, and patients with well-controlled *versus* uncontrolled intrahepatic tumors. Our findings suggest that HT, especially in combination with sorafenib, may be a promising approach in a subgroup of patients.

Among patients with extrahepatic metastases from HCC, those with pulmonary metastases had a better prognosis than those with metastases at other sites (e.g., bone and adrenal gland), as reported previously by our group [16–17]. In this study the most common cause of death was brain hemorrhage, rather than lung failure. Pulmonary metastases in HCC patients are seldom the cause of death, but they suggest the presence of brain metastasis, which can result in brain hemorrhage (associated with a median survival of only 1-3 months) [18]. Hence, we believe that uncontrolled intrahepatic tumors are the most critical problem to address in HCC, and aggressive treatment of intrahepatic tumors contributed in part to the improved OS observed in patients who received sorafenib in this study.

**Figure 5:**
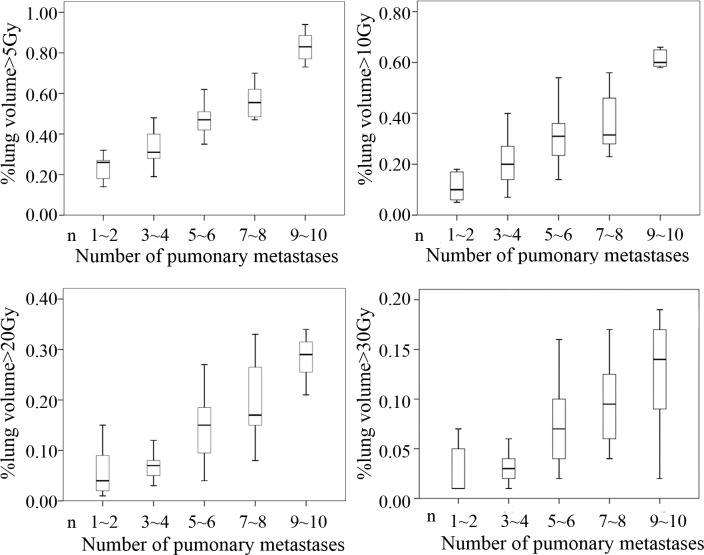
Conformal avoidance for HT V_20_ and V_30_ of lung dose-volume parameters using HT were > 30% and > 20%, respectively, whereas V_5_ and V_10_ were relatively high.

Because most of the patients in this study had not previously received systemic chemotherapy and had no underlying lung diseases, we were able to deliver a total dose of 50 Gy to the lung lesions, with V_20_ limited to 30%. No severe complications occurred, except for radiation esophagitis and pneumonitis (grade 1-2). We were able to minimize critical injury to the lungs through the use of four-dimensional computed tomography (4D-CT) simulation to track tumor motion during free breathing. In a study of patients with stage III inoperable non-small-cell lung cancer, the dose distribution of HT was shown to spare the lungs and spinal cord with reasonable tumor dose homogeneity [19], indicating that lung dose-volume measures are considerably lower for HT than for 3D-CRT. We evaluated dose distribution in the present study and found similar values for dose-volume parameters for multiple lesions. As shown in Figure 5, V_20_ and V_30_ for ≥ 7 lesions were less than 30% and 20%, respectively, although V_5_ and V_10_ were relatively high. However, in our experience, it is difficult to deliver adequate doses to all lesions without exceeding lung tolerance in patients with ≥ 10 lesions. In the dosimetric analysis, particular emphasis must be placed on sparing of the lung and preventing radiation pneumonitis. Although HT has superior dose distribution and normal tissue sparing, the cost was no more than that of conventional radiotherapy because hypofractionated radiotherapy was used, and the entire procedure was completed in only 1 or 2 weeks.

Our results showed no significant relationships between clinicopathologic features and response to radiotherapy. Although the addition of sorafenib delayed time to progression, there was no evidence of its effect on tumor response. Sorafenib acts by inhibiting tumor cell proliferation and tumor angiogenesis and increasing the rate of apoptosis [20–21]. Our results suggest that its inhibition of tumor cell proliferation and angiogenesis may be greater than its ability to increase the rate of apoptosis.

The present study has several limitations. First, although this is one of the largest studies of HT combined with sorafenib for the treatment of pulmonary metastases from HCC from a single institution, the sample size is small; therefore, multicenter studies are needed to confirm these results. Second, the retrospective nature of this study is a major limitation. Last, patients in the study may not be representative of the general patient population with pulmonary metastases from HCC, because our study included five patients with other extrahepatic metastases besides lung lesions. Future studies should be conducted to optimize the clinical application of tomotherapy.

## MATERIALS AND METHODS

### Study design and patient population

This was a retrospective, single-center study conducted from October 2011 to October 2014. To minimize selection bias, all eligible patients were consecutively enrolled into the study and treated according to the protocol. HCC was diagnosed based on histology (surgical or transplantation specimen, or needle biopsy) before the initial treatment for intrahepatic tumors. The diagnosis of lung metastases from HCC was confirmed by at least two radiologists and oncologists based on lung CT scans. Well-controlled intrahepatic primary tumors were defined as follows: (1) after the first treatment for HCC, no new lesions were detected by follow-up enhanced CT, magnetic resonance imaging (MRI), or both during the remaining treatment period, or (2) after intrahepatic recurrence, the patient underwent transcatheter arterial chemoembolization or radiofrequency ablation, and lipiodol was deposited in the entire intrahepatic tumor, or the tumor was destroyed within the zone of ablation, and follow-up enhanced CT or MRI did not show any new lesions even at the edge of primary tumors during the treatment period. In all other cases, the patients were regarded as having uncontrolled primary tumors. Patients included in the validation cohort had well-controlled intrahepatic tumors, and all patients in this present study had an Eastern Cooperative Oncology Group (ECOG) status of 0-2.

Inclusion criteria were eligibility for radiation therapy; complete records of clinicopathologic characteristics; unsuitability for pulmonary resection or refused resection; no prior thoracic irradiation; no active systemic, pulmonary, or pericardial infection; absence of mediastinal node metastasis based on CT and positron emission tomography/CT; no coexisting lung disease; longest tumor diameter < 5 cm; and < 10 metastatic lesions. The protocol was approved by the local institutional review boards and ethics committees, in accordance with national and international guidelines. Signed informed consent forms were obtained from all patients.

A total of 195 pulmonary metastatic lesions were detected in the enrolled 45 patients. In addition, right adrenal gland metastases in 2 patients and bone metastases in 3 patients (ribs, *n* = 2; right scapula, *n* = 1) were detected at the same time as pulmonary metastases. These tumors were also treated with HT and managed until the last follow-up examination.

Sorafenib was recommended in our institution, especially during radiotherapy, based on the drug's effectiveness in increasing OS in metastatic HCC. However, some patients discontinued sorafenib treatment because of its cost. The 45 patients were divided into two treatment groups: HT combined with sorafenib (*n* = 23) or HT only (*n* = 22). In the combination treatment group, 15 patients received 400 mg sorafenib twice daily (i.e., 800 mg/day), and 8 patients received a smaller dose (200 mg sorafenib twice daily or 400 mg/day) because of AEs. The median duration of treatment with sorafenib was 54.0 ± 15.7 days (range, 25-400 days).

### Treatment

**Figure 6:**
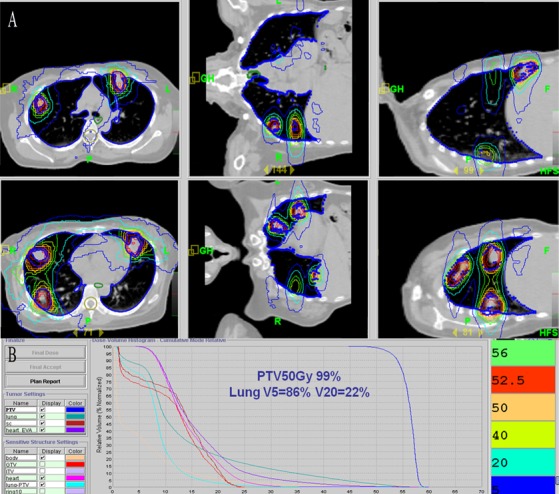
Dose distribution and dose-volume histogram for HT **A.** Representative dose distribution for a patient with multiple lesions *via* anterior-posterior parallel-opposed fields on axial images in bilateral lobes based on two isocenters. **B.** Mean dose-volume histograms for gross target volume (GTV), planning target volume (PTV), healthy tissue, and organs at risk (OARs).

Treatment with HT for metastatic lesions is described in detail elsewhere [2]. In brief, patients underwent planned CT scans in the treatment position. CT scans (3 mm thick) were obtained from the lower end of the cricoid cartilage to the lower edge of the liver, and the resulting images were imported into the 3D planning system (CMS XiO Treatment Planning System, Elekta Medical Systems). Gross tumor volume (GTV) was defined as the volume of a macroscopic tumor. The motion of the tumors and other internal organs during free breathing was measured by 4D-CT simulation. The extent of pulmonary tumors was delineated on pulmonary windows. Internal target volume, generated by the expansion of GTVs during the four phases of each respiratory cycle from the 4D-CT scan, included a margin to account for patient movement. To compensate for daily set-up errors, the planning target volume (PTV) was extended 0.4 cm. The organs at risk, such as the spinal cord and lungs, were contoured. Dose constraints for PTV were as follows: (1) 95%-110% of the prescribed dose was delivered to the entire PTV, (2) the lung volume receiving ≥20 Gy (lung V_20_) was limited to 30% in all patients, and (3) none of the PTVs received ≥115% of the prescribed dose. Figure 6A shows the dose distribution for one patient from the delivery of 50 Gy to pulmonary lesions, and Figure 6B shows the average dose-volume histogram for GTV, PTV, healthy tissue, and organs at risk. A total dose of 50 Gy in 5 or 10 fractions was prescribed at the physician's discretion based on the patient's general condition, number of lung lesions, and radiation dose delivered to normal organs.

The patients with intrahepatic tumors underwent radiofrequency ablation once during radiotherapy (*n* = 4), transcatheter arterial chemoembolization (*n* = 7), or both modalities (*n* = 1).

### Follow-up

We collected data on the treatment administered for the primary hepatic tumors and patients' baseline characteristics before the start of radiotherapy (gender, age, α-fetoprotein level, number of pulmonary metastases, intrahepatic tumor status, metastases in sites other than the lung, and ECOG status). Data of patients who were alive without disease progression or dead without documented progression at the last observed follow-up were censored. Clinicopathologic variables are detailed in Table 3.

**Table 3:** Clinicopathologic profiles of 45 patients with pulmonary metastases from hepatocellular carcinoma

		Patients	%
Gender			
	Male	38	84.4
	Female	7	15.6
Age			
	≥60	14	31.1
	<60	31	68.9
PDR(months)			
	≥6	14	31.1
	<6	31	68.9
PmFI(months)			
	≥12	24	53.3
	<12	21	46.7
AFP of pre-RT			
	≥20	36	80
	<20	9	20
Viral hepatitis			
	Present	42	93.3
	Absent	3	6.7
No. of metastases			
	*n* ≤ 3	18	40
	*n* > 3	27	60
Maximum size of the metastatic lesions(cm)			
	≤3	34	75.6
	> 3	11	24.4
Intra-hepatic tumor			
	Active	12	26.7
	Inactive	33	73.3
Therapeutic models for liver tumor			
	Resection	24	53.3
	TACE	3	6.7
	Liver transplantation	18	40
Sorafenib			
	No	22	48.9
	Yes	23	51.1
ECOG performance status			
	0-1	39	86.7
	2	6	13.3
Other metastasis except lung			
	No	40	88.9
	Yes	5	11.1

Abbreviations: PDR, period from the detection of pulmonary metastases to radiotherapy; PmFI, Pulmonary metastases-free interval; AFP, α-Fetoprotein; TACE, Transarterial chemoembolization; ECOG, Eastern Cooperative Oncology Group.

The interval between the initial therapy for primary hepatic tumors and detection of pulmonary metastases (pulmonary metastases-free interval) for patients in this study ranged from 1.0 to 72.3 months (median, 16.0 ± 5.5 months). The interval between the detection of pulmonary metastases and radiotherapy for lung lesions ranged from 0.1 to 28.2 months (median, 3.7 ± 1.9 months).

All patients underwent contrast-enhanced CT scans 1 week before the start of HT and 1 month after the completion of HT. Follow-up exams at 3-month intervals included contrast-enhanced CT, measurement of serum tumor markers, and ultrasonography of the liver. The median follow-up period was 28.9 ± 12.3 months (range, 15.5-45 months). At the time of the last follow-up (October 24, 2015), a total of 29 (64.4%) patients had died.

### Evaluation of response and toxicity

The treatment response of the targeted lesions was evaluated by contrast-enhanced CT scan using the guidelines of the Response Evaluation Criteria in Solid Tumors Group [22]. Patients with CR (complete disappearance of all assessable lesions) or PR (≥30% reduction in the sum of the maximum diameters of all measurable lesions) were considered responders. Patients with SD (<30% reduction to <20% increase in the sum of the maximum diameters of all measurable lesions) or PD (≥20% increase in the areas of the original measurable lesions or appearance of a new lesion) were considered nonresponders. Lesions within an area of radiation pneumonitis or fibrosis that did not change in size were considered an indication of SD. Response to radiotherapy was ascertained by two radiologists independently comparing the CT images obtained before and after treatment.

Complete blood cell counts and routine chemistry tests were performed once a week during the course of treatment. Acute toxicities such as radiation pneumonitis, esophagitis, skin reactions, and hematologic toxicity were assessed. In addition, sorafenib-induced AEs such as increased levels of aspartate transaminase and alanine transaminase, hand-foot skin reaction, and anorexia were analyzed. Toxicity was assessed using the Common Terminology Criteria for Adverse Events (CTCAE v3.0) [23].

### Further validation of combination treatment

For further validation, we compared the OS rates of patients with pulmonary metastases from HCC treated with a combination of sorafenib and radiotherapy (Co group, *n* = 18), radiotherapy only (Rt group, *n* = 15), or sorafenib only (S group, *n* = 18). The patients in the Co and Rt groups were part of the 45 patients enrolled in this study and had no active hepatic tumors. Data on patients receiving sorafenib only were collected between 2007 and 2011; in these patients, complete control of the intrahepatic tumors was achieved; diagnosis of lung metastases and other selection criteria were equivalent to that of the 33 patients in the Co and Rt groups. The median duration of sorafenib treatment was 62.0 ± 15.8 days (range, 33.0-325.0 days). Median follow-up for this cohort was 35.0 ± 11.2 months (range, 11.0-56.0 months). Clinicopathologic characteristics of the three groups are described in Supplementary Table S2.

### Statistical analyses

PFS was defined as the time from the initiation of radiotherapy to any type of metastatic lesion progression or new lesion appearance. OS was defined as the time from the detection of lung lesions to death from any cause or the last follow-up appointment. Relationships between clinical variables were assessed using Pearson's chi-square test or Fisher exact test, whichever was appropriate. Survival curves were calculated using the Kaplan-Meier method, and univariate analysis was performed using the log-rank test. Multivariate analysis was performed using a forward-stepwise Cox regression model to explore associations between clinical variables and PFS or OS. A P value less than 0.05 was considered statistically significant. Data analyses were performed using SPSS version 16.0 for Windows.

## SUPPLEMENTARY MATERIAL


