# Supplementary material for: Simultaneous multitarget radiotherapy using helical tomotherapy and its combination with sorafenib for pulmonary metastases from hepatocellular carcinoma
Source: Oncotarget. 2016 May 14;7(30):48586–99. doi: 10.18632/oncotarget.9374 (PMC5217040; doi:10.18632/oncotarget.9374)
Supplement: Supplementary file 1 [file oncotarget-07-48586-s001.pdf]

Simultaneous multitarget radiotherapy using helical tomotherapy and its combination with sorafenib for pulmonary metastases from hepatocellular carcinoma

Supplementary Material

Supplementary Table S1. Baseline characteristics of groups with response to treatment of pulmonary metastasis

Parameters		CR+PR	SD+PD	*P*
Gender				
	Male	25	13	0.674
	Female	5	2	
Age				
	≥60	9	5	0.492
	<60	21	10	
PDR(months)				
	≥6	10	4	0.988
	< 6	20	11	
PmFI(months)				
	≥12	15	9	0.421
	<12	15	6	
AFP of pre-RT				
	≥20	25	11	0.355
	<20	5	4	
Viral hepatitis				
	Present	28	14	0.746
	Absent	2	1	
No. of metastases				
	n≤3	15	3	0.127
	n>3	15	12	
Maximum size of the metastatic lesions(cm)				
	≤3	21	13	0.198
	> 3	9	2	
Intrahepatic tumor status				
	Active	6	6	0.279
	Inactive	24	9	
Therapeutic models for liver tumor				
	Resection	16	8	0.410
	TACE	1	2	
	Liver transplantation	13	5	
Sorafenib				
	No	14	8	0.463
	Yes	16	7	

ECOG performance status				
	0-1	26	13	0.375
	2	4	2	
Other metastasis except lung				
	No	27	13	0.826
	Yes	3	2	

Abbreviations: PmFI, Pulmonary metastases-free interval; AFP, α-Fetoprotein; PDR, period from the detection of pulmonary metastases to radiotherapy; TACE, Transarterial chemoembolization; ECOG, Eastern Cooperative Oncc Group. *P* values were obtained in Chi-square test.

Supplementary Table S2. Characteristics of patients in 3 cohorts

		Radiotherapy	Sorafenib	Combination
Gender				
	Male	14	16	16
	Female	1	2	2
Age				
	≥60	5	5	4
	<60	10	13	14
PmFI(months)				
	≥12	8	12	11
	<12	7	6	7
AFP of pre-RT				
	≥20	11	14	15
	<20	4	4	3
Viral hepatitis				
	Present	14	18	17
	Absent	1	0	1
No. of metastases				
	n≤3	4	10	8
	n>3	11	8	10
Maximum size of the metastatic lesions(cm)				
	≤3	11	13	12
	> 3	4	5	6
Therapeutic models for liver tumor				
	Resection	6	11	12
	TACE	0	0	0
	Liver transplantation	9	7	6
ECOG				
	0-1	13	14	15
	2	2	4	3
Other metastasis except lung				
	No	15	18	17
	Yes	0	0	1

Abbreviations: PmFI, Pulmonary metastases-free interval; AFP, α-Fetoprotein; TACE, Transarterial chemoembolization; ECOG, Eastern Cooperative Oncology Group.
